# Stakeholder engagement in genetics research development in diverse urban communities in the Midwestern United States: process and considerations

**DOI:** 10.3389/fmed.2026.1816840

**Published:** 2026-05-08

**Authors:** Sally E. Jensen, Shannah Mwanda, Clarissa Huard, Christine Drogan, Susan G. Fisher, Sonia Kupfer, Elbert S. Huang, Sean David

**Affiliations:** 1 Health Outcomes Research Collaborative, Research Institute, Endeavor Health, Evanston, IL, United States; 2 Department of Family Medicine, Endeavor Health, Evanston, IL, United States; 3 University of Chicago, Chicago, IL, United States

**Keywords:** community engagement, genetics, genomics, primary care, research development, stakeholders

## Abstract

**Introduction:**

The inclusion of individuals from diverse backgrounds is essential to enhancing the generalizability and interpretability of genetic testing results. It is imperative to foster stakeholder engagement at all steps in the research process in diverse communities, given past and present experiences of marginalization and discrimination in healthcare research. A stakeholder engagement workshop was convened to provide input on study design and community priorities for genetic research in diverse communities.

**Methods:**

Seventeen stakeholders including primary care providers (PCP) and patient advocates from diverse communities provided anonymous quantitative (ranking exercises) and qualitative (group discussion) input during a 2-h web-based workshop. Stakeholders discussed the following topics: prioritization of conditions to include in community-based genetic screening research and considerations for genetic research development and design in diverse communities. Emergent themes were noted and summarized to inform future research development.

**Results:**

Community stakeholder discussion themes included: rationale for conditions to include in community-based genetic screening, strategies for community trust-building, the importance of actionability and support following results, provision of informational support related to genetic testing privacy issues and protections, and inclusion of genetic testing education for both PCPs and community members.

**Discussion:**

During this workshop, stakeholder input addressed considerations across the research continuum from study conceptualization to the application of findings in diverse community settings. Stakeholders’ comments provide valuable insights on the development of a genetic screening program that incorporates their input from the beginning to beyond the completion of the study period.

## 1 Introduction

Genetic conditions encode or affect the lifetime risk of chronic, sometimes severe disorders such as hereditary cancer, cardiovascular disease, and multiple organ system syndromes. Genetic conditions, including both more common and rare diseases, affect about one in ten people in the United States (U.S.) population (1). Given the public health impact of genetic conditions, the Centers for Disease Control and Prevention (CDC) identified tiers of conditions diagnosable through genetic screening to prioritize population testing strategies using a range of approaches (2). A scoping review of population-based genetic screening programs (3) identified a limited range of large-scale (tens of thousands of patients or more) genetic screening programs across the U.S. and internationally that have used a variety of sequencing platforms and systems (state- or province-wide, national, or health system-based). These programs have enabled the testing of millions of individuals, from newborn screening for childhood-onset diseases to adult screening for actionable conditions (3).

However, robust, population-scale testing for actionable genetic disorders has not yet been achieved in the U.S. – a significant opportunity gap in public health. Recognizing this gap, the U.S. National Human Genomic Research Institute convened a webinar (4) in which they issued requests for applications to form a Population Genomic Screening in Primary Care multi-site clinical trial network (5). As one of the applicant organizations, our team, spanning multiple sites across the greater metropolitan Chicago (Illinois, U.S.) area, formed a community advisory board and invited widespread provider and patient advocate input to guide patient-centered genetic screening research to optimize participation by primary care teams serving underserved and minority communities.

Patient-centeredness is increasingly prioritized in genetics research, with an emphasis on stakeholder engagement throughout the research trajectory (6). Stakeholders have been defined as “an individual, group of individuals, or organization that has a vested interest or a stake that can affect or be affected by a course of action,” (7). Stakeholder engagement is extremely important when considering genetic screening in diverse communities with historical and contemporary experiences of marginalization and discrimination in healthcare and research (8, 9). Community-based genetic testing can offer increased access to risk information with actionable implications in disease prevention, treatment, and decision-making (10). To ensure that community-based genetics research is culturally sensitive, community stakeholder engagement throughout the process is crucial. Several currently existing programs support community stakeholder engagement in genetic research with diverse communities. In the U.S., the NIH Build Up Trust Challenge provided funding for strategies that increase research participation through trust-building and engagement with underrepresented communities^1^, including one project that seeks to bring the benefits of genomics to diverse Bronx (New York City) communities^2^. In Australia, the QIMR Berghofer Medical Research Institute utilized stakeholder engagement to develop best practice guidelines for conducting genomic research with Aboriginal and Torres Strait Islander communities (11). Worldwide, the biotech company Variant Bio has prioritized community-engaged research partnerships that give back to the partner community through benefit-sharing plans specific to the community’s needs and priorities, and ensuring that results are first disseminated to the partner communities^3^. The broad objective of this research was to identify best practices for achieving equitable and effective implementation of population genomic screening in diverse primary care practices and patient populations. Consistent with these initiatives, the purpose of this workshop was to engage community stakeholders in diverse urban communities to help prioritize additional health conditions for inclusion in a genetic screening study and to inform future genetic research development.

## 2 Materials and methods

The following “preparatory to research” work laid the foundation for future grant-generating programs to design, implement, and study optimal approaches to community-based primary care genetic screening in the greater metropolitan Chicago area. We formed the Chicagoland Equitable Population Genomic Screening in Primary Care (COALESCE) clinical group, a collaboration between the University of Chicago and Endeavor Health, to conduct genomic screening in diverse patients through primary care clinics across the Chicago metropolitan region. COALESCE is multidisciplinary in its composition, including investigators with backgrounds in primary care medicine, genetic medicine, genetic counseling, epidemiology, and social science. COALESCE was supported locally by this stakeholder advisory board and collaborations with the Chicago Chronic Condition Equity Network (P50MD017349), its Community-Based Research Network, our Clinical and Translational Science Award (CTSA)’s (UL1TR002389) Community & Collaboration core, and the Institute for Population & Precision Health at the University of Chicago. Our COALESCE team convened a workshop with patient advocates, community partners, and primary care providers (PCPs) to inform the prioritization of genetic conditions for screening and to develop an implementation plan aligned with the values of our communities and the PCPs who serve them.

The Endeavor Health Institutional Review Board assigned a determination of Not Human Subjects Research for this project (Protocol # IRB2025-0091); therefore, informed consent was not required. All workshop data were anonymous and not directly linked to individuals.

### 2.1 Participants

Eligible stakeholders were identified by senior members of the team with established backgrounds in community-based genetic research. Stakeholders were invited via email to participate in the workgroup based on their experience as either leaders of community-based organizations, primary care physicians working in diverse communities, or patient advocates with lived experience.

### 2.2 Procedures

Stakeholders participated in a 2-h web-based workshop facilitated by a primary care physician and a genetic counselor. The workshop provided: (1) an overview of the rationale for the development of a community-based genetic testing program of research, and (2) the opportunity for stakeholders’ anonymous quantitative and qualitative input through a combination of priority ranking exercises and group discussion.

Stakeholders were first provided with an overview of the workgroup purpose. Facilitators explained the Centers for Disease Control and Prevention (CDC) designation of three “Tier 1” conditions (Hereditary Breast and Ovarian Cancer (HBOC), Lynch Syndrome, and Familial Hypercholesterolemia) that are cost-effective to include in primary, population-based genetic screening based on evidence of reduced morbidity and mortality resulting from appropriate preventive interventions (12). Stakeholders were then introduced to 10 genetic conditions identified by the American College of Medical Genetics and Genomics (ACMG) as reportable secondary findings for screening (13). They were asked to rank the four highest priority conditions from the ACMG list using REDCap by responding to the question, “Please choose your top choice for inclusion,” followed by, “Please choose your second choice for inclusion. Do not select the disease you chose for question 1”; “Please choose your third choice for inclusion. Do not select the disease you chose for question 1 or 2”; and “Please choose your fourth choice for inclusion. Do not select the disease you chose for question 1, 2, or 3.” Group discussion about their rationale for rankings followed the initial ranking exercise. Stakeholders could then change their rankings based on the discussion using the same questions asked during the first ranking exercise. Finally, stakeholders were asked to engage in a discussion related to genetic research design and development (Table 1). Three team members took discussion field notes but did not participate in the discussion.

**TABLE 1:** Stakeholder workshop discussion questions.

Priority of conditions for genetic screening	Study design and logistics
1. Why did you choose your first ranked disease?	1. How can we earn trust from diverse communities?
2. What were you prioritizing in making your decisions?	2. Do you have any feelings about genetic testing and results? How can the experience be made more meaningful?
3. Were there any diseases that you left out of your top four that you struggled with? Why?	3. What are some of your priorities or that of your community members in participating in genomic research?
4. Were there any diseases that you think should not be included?	4. How should we feasibly and effectively educate/train PCPs? How would this best work for your practice?
5. Did we leave any diseases out that you would like to see included?	5. How, when, and where should we approach people to participate and collect samples for genetic testing?
–	6. What do you think about the delayed release of some additional, less actionable results?

### 2.3 Data analysis

Responses to the ranking questions were analyzed using frequency distributions. Stakeholder discussion input based upon field notes was summarized according to themes using a descriptive-interpretive analysis approach (14).

## 3 Results

### 3.1 Stakeholder characteristics

Seventeen stakeholders (including PCPs, patient advocates, community advisory council members, and research staff) participated in the workshop. No sociodemographic data were collected in order to preserve anonymity.

### 3.2 Prioritization of conditions for genetic testing

**FIGURE 1:**
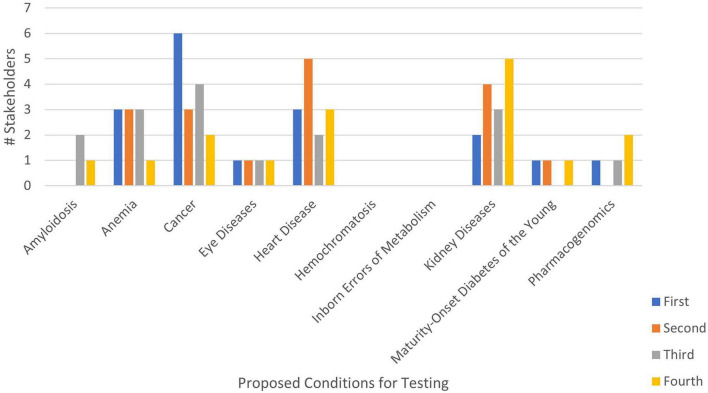
Genetic condition rank choice by number of stakeholders during first ranking exercise.

Members ranked 10 genetic conditions to include in addition to Tier 1 conditions before and after the prioritization discussion. Table 2 and Figure 1 present the first ranking exercise results.

**TABLE 2:** Initial (pre-discussion) condition importance rankings (*N* = 17).

ACMC candidate condition	Overall rank	Ranked first in priority, *n* (%)
Cancer	1	6 (35.3%)
Anemia	2	3 (17.6%)
Heart diseases	3	3 (17.6%)
Kidney diseases	4	2 (11.8%)
Eye diseases	5	1 (5.9%)
Maturity-onset diabetes of young	6	1 (5.9%)
Pharmacogenomics	7	1 (5.9%)
Amyloidosis	8	0
Hemochromatosis	8	0
Inborn errors of metabolism	8	0

Stakeholders were asked to rank their four highest priority conditions. This table presents the frequency of receiving a ranking of first in priority by condition. Overall Rank was determined by the frequency with which each condition was ranked first in priority.

During discussion, stakeholders described actionability as a priority for rankings, expressing skepticism about testing for conditions with no actionable interventions. Stakeholders emphasized consideration of the prevalence and impact of a condition in specific communities. They recommended addressing conditions with the greatest inequities, latest detection rates, and most treatment disparities. Stakeholders also prioritized conditions with greater awareness among community members. Other ranking priorities included personal/family history and less-researched conditions.

**FIGURE 2:**
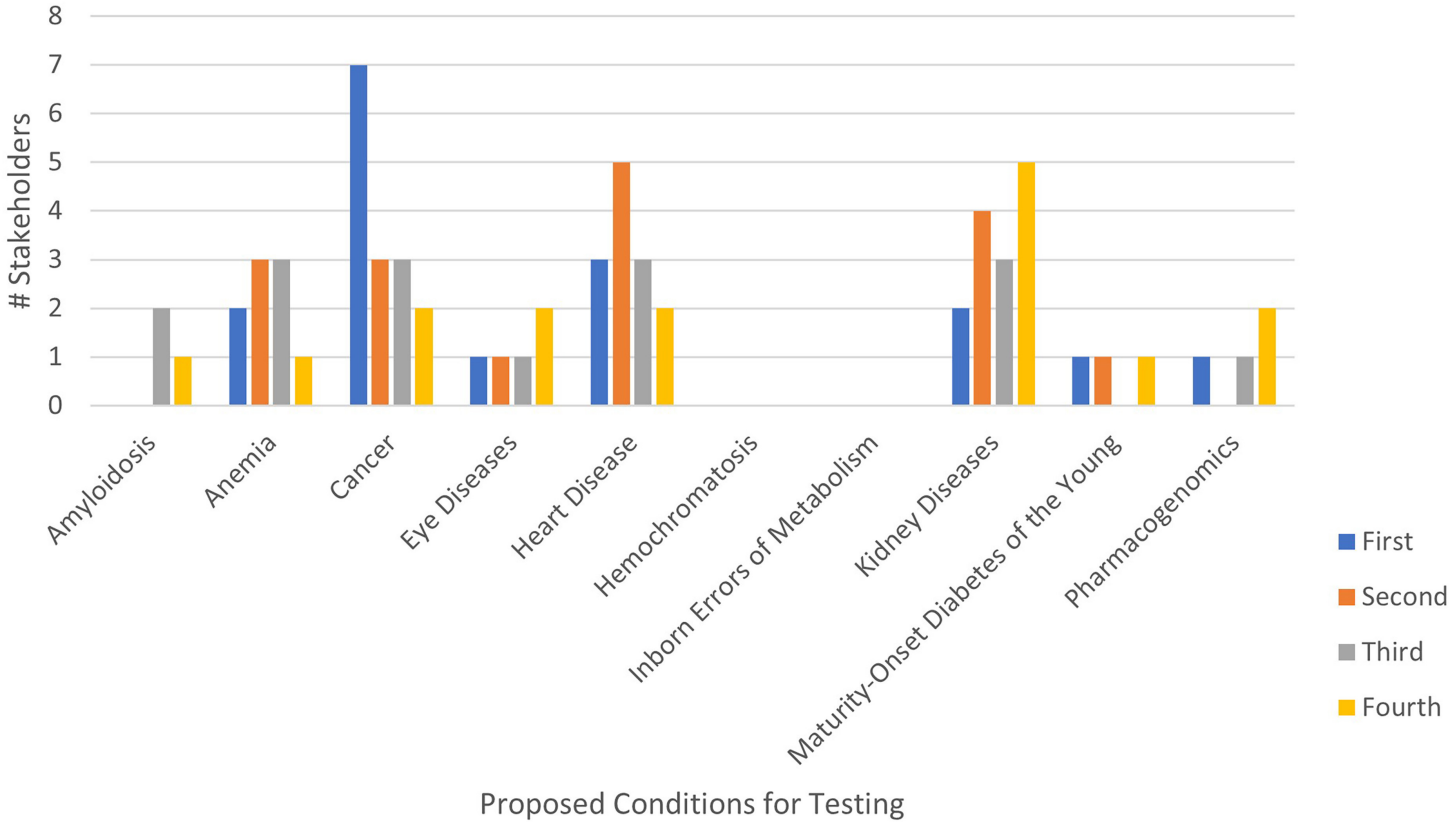
Genetic condition rank choice by number of stakeholders during second ranking exercise.

Following the ranking discussion, stakeholders were offered the opportunity to change their rankings (Table 3 and Figure 2). The overall order of the conditions ranked first in priority did not change substantially from the first to the second ranking, with the exception of heart diseases and anemia. The elevated prioritization of heart diseases over anemia in the second ranking may have been influenced by questions about the availability of actionable anemia interventions during the discussion.

**TABLE 3:** Follow-up (post-discussion) condition importance rankings (*N* = 17).

ACMC candidate condition	Overall rank	Ranked first in priority, *n* (%)
Cancer	1	7 (41.2%)
Heart diseases	2	3 (17.6%)
Anemia	3	2 (11.8%)
Kidney diseases	3	2 (11.8%)
Eye diseases	5	1 (5.9%)
Maturity-onset diabetes of young	5	1 (5.9%)
Pharmacogenomics	5	1 (5.9%)
Amyloidosis	8	0
Hemochromatosis	8	0
Inborn errors of metabolism	8	0

Stakeholders were asked to rank their four highest priority conditions. This table presents the frequency of receiving a ranking of first in priority by condition. Overall Rank was determined by the frequency with which each condition was ranked first in priority.

### 3.3 Considerations for future genetic research design and development

#### 3.3.1 Trust

Trust was a theme that emerged throughout the group discussion, but was particularly salient in the context of discussion questions, “How can we earn trust from diverse communities?” and “Do you have any feelings about genetic testing and results? How can the experience be made more meaningful?” Stakeholders emphasized directly acknowledging historical and current discrimination pertaining to genetic research. They stressed the example of Henrietta Lacks, its impact on trust in the community, and concern about lack of information regarding dissemination and sharing of genetic results or how the data may be used in the future.

Stakeholders also highlighted the need for community connection and liaisons with whom community members feel comfortable and safe. They differentiated between researchers who may be perceived as authority figures and individuals who have authentic relationships with community members. They also encouraged diversity in the research team, noting the potential benefits of cultural concordance between researchers/clinicians and community members.

Relationship-building and patient advocacy also emerged as important trust themes. Stakeholders encouraged speaking directly with community partners, actively engaging with them, and learning about their most impactful health concerns. They stated that genetic information belongs to the patients, who need ownership of the information and its use. Discussion addressed whether all genetic data should be shared with patients and how the management of genetic results could impact trust, particularly with respect to delayed release of results.

#### 3.3.2 Actionable steps

Actionable steps and the need for support following results was a prominent workshop theme, particularly when discussing the following questions: “Do you have any feelings about genetic testing and results? How can the experience be made more meaningful?” “What are some of your priorities or that of your community members in participating in genomic research?” and “What do you think about the delayed release of some additional, less actionable results?” Stakeholders discussed the limitations of tests and the need to provide clear information about what the tests can reveal and the limitations of the results to help manage expectations. They also raised the question of what can/will be done with testing results and what that might mean for their families. Discussion of actionable steps also centered around the sharing of results, with differing stakeholder opinions about sharing non-actionable results. For example, some stakeholders reported that all results should be shared whether or not they are urgent or actionable, while others voiced concern that if the result is not actionable, its disclosure may not be desired by patients.

The imperative to provide support after results was a predominant discussion theme. Stakeholders discussed the role of the institution in supporting patients after genetic testing results as an institutional duty to ensure equal access to follow-up care. They suggested embedding patient navigators in the clinic to minimize PCP burden and assure follow up about actionable results. They also recommended developing primary care workflows that allow for longer appointment times and clear paths to connect patients with actionable results to appropriate clinical specialists.

#### 3.3.3 Community priorities

Addressing health disparities emerged as a prominent discussion theme, particularly with respect to the diagnosis and treatment of diseases in African American communities. Discussion of community priorities primarily arose in response to the question, “What are some of your priorities or that of your community members in participating in genomic research?” Stakeholders emphasized addressing the most prevalent diseases in their communities, tailoring genetic research to diverse populations and individuals’ needs, and focusing on health concerns and family histories associated with genetic research in the community.

#### 3.3.4 Privacy

Another prominent discussion theme centered around transparency and clarity about current genetic testing legal protections. Privacy emerged as a theme across the discussion, but particularly in response to the questions, “How can we earn trust from diverse communities?” “Do you have any feelings about genetic testing and results?” and “What are some of your priorities or that of your community members in participating in genomic research?” Stakeholders identified privacy as a substantial barrier to testing, given the lack of protection from discrimination for long-term disability or life insurance based on genetic test results. They remarked that providing information related to the United States Genetic Information Non-discrimination Act of 2008 (GINA), Public Law 110–233, and/or legal aid resources to community members may be beneficial.

Stakeholders also expressed concerns about assurance that DNA samples are being tested for the agreed upon purpose. Several stakeholders spoke about the documentation of genetic testing results in medical records and questioned whether a model could be implemented to ensure that genetic results from research would not be documented in medical records.

#### 3.3.5 Education

Although this theme emerged during discussion of the question, “How should we feasibly and effectively educate/train PCPs? How would this best work for your practice?” the discussion also addressed education for patients. Stakeholders noted that variability in health literacy levels might affect understandability of genetic testing and recommendations. They suggested pairing patients with a genetic counselor/navigator to provide understandable lay language explanations to prevent individuals from feeling overwhelmed. Stakeholders also discussed the importance of knowledgeable PCPs, citing their frontline role in clinical care, established connections with patients in the community, and ability to answer community members’ questions– all of which can foster engagement. Stakeholders also advocated for PCP cultural sensitivity training, differentiating it from diversity training. They emphasized that PCP education about different cultural perspectives on genetic testing would help enable demonstration of cultural competence in the community setting.

#### 3.3.6 Logistics of community engagement in genetic research

The question, “How, when, and where should we approach people to participate and collect samples for genetic testing?” prompted a discussion about logistical considerations for community engagement, not only in terms of clinical screening, but also related to community partnership in genetic research more broadly. Whereas the aforementioned discussion themes addressed community engagement in genetic testing research design at a conceptual level, stakeholders’ comments related to logistics were more of a detailed, tangible nature. Stakeholders suggested several options for trainings to facilitate community and clinician engagement, including webinars, panels, workshops, and lectures. Discussion also addressed offering continuing education credits for PCPs, with assistance from community-based organizations and nursing schools in curriculum development. Stakeholders recommended utilizing spaces perceived as “safe” by community members to introduce genetic research participation opportunities. Stakeholders encouraged establishing partnerships with local clinics, representation at community health and townhall events, and advertising locally to assist with community member recruitment. Some discouraged restricting outreach to locations such as business establishments and religious congregations where community members regularly socialize. Stakeholders also addressed access issues, such as transportation reimbursement, assistance with obtaining Medicaid, and provision of informational resources to community members. Several stakeholders noted that many patients receive healthcare at smaller community clinics and specialized organizations and recommended a conscientious approach with community members who are underinsured and/or lack continuity of care.

## 4 Discussion

This research development initiative involved a community stakeholder workshop to provide input on the design and development of future genetic research in diverse communities. In addition to logistics, stakeholder discussion centered around rationale for conditions to include in community-based genetic screening, trust-building, actionability, genetic testing privacy issues, and education. Given that genetics research in diverse communities involves more than just recruitment (9), the workshop discussion themes addressed numerous considerations across the research continuum from study conceptualization to the application of findings in diverse community settings. Throughout the workshop, trust emerged as essential to community-based genetic research development. Stakeholder discussion highlighted the fact that building trust is not simple; rather, it is an ongoing, long-term, iterative, mutually respectful process (9) as opposed to a linear process wherein trust is an endpoint that is “achieved.”

It is noteworthy that stakeholder feedback also addressed the clinical implications of genetic testing research after the completion of a study. Given potential barriers in access to clinical care that might be warranted based on a genetic result, research should incorporate plans for providing support to enable appropriate clinical follow-up during the initial research planning stages. This may involve securing “buy-in” from institutions and arrangements to address the costs of clinical follow-up that may result from participation in the study if an actionable result is found. Interestingly, the issue of Clinical Laboratory Improvement Amendments (CLIA) regulation (15) did not arise during the workshop. This may be due to the fact that candidate genetic conditions for testing only included testing that would be done in CLIA-certified laboratories or the fact that stakeholders may not have been familiar with CLIA regulations pertaining to genetic testing for clinical purposes. Given its important role in terms of actionability, ensuring that genetic tests with clinical utility are properly carried out according to CLIA regulations remains an essential aspect of genetic testing research planning and education.

Stakeholders’ emphasis on actionability related to the provision of clinical follow-up to actionable results received as part of research was seemingly at odds with comments related to privacy concerns about documentation of genetic research results in medical records. This discrepancy may reflect an incomplete differentiation between research and clinical practice, which may be likely to arise in genetic research that is implemented into clinical settings, such as primary care. Stakeholders’ somewhat contradictory comments related to actionability and privacy highlight the value of incorporating stakeholder input early in the research development process to identify and address such gaps in understanding to ensure that the overall purpose is understandable and aligned with community needs and priorities.

Perspectives differ in the optimal level of stakeholder engagement when considering community-based research development. For example, community-based participatory research (CBPR) consists of equitable, direct involvement of various types of stakeholders (e.g., community members, researchers, healthcare providers etc.) in all phases of the research process; namely, all stakeholders share in decision-making and ownership of the research project (16, 17). The level of community member engagement can vary across a spectrum from less engaged (e.g., outreach to community members) to most engaged (e.g., shared leadership of the project between community members and researchers) (18). CBPR is predicated upon the equal valuation of the unique perspectives of all stakeholder types in an effort to address health and research inequities and to engender meaningful research for the population which it seeks to serve (16). For this reason, the CBPR framework is often implemented in research related to health equity and diverse communities (16). Despite many benefits, CBPR challenges include the time, flexibility, training needs, cost, and the institutional support required (19). The cost, time, and resources required to support a full adoption of all CBPR principles was not feasible for the proposed scope of this genetic screening research program. However, Collins et al. (16) emphasize that the principles of CBPR do not require “absolute” or “comprehensive” implementation and should be flexibly modified as needed for diverse community-based studies in an iterative manner. In recognition of the valuable insight and contributions community members can provide in developing genetic screening research in diverse urban communities, this stakeholder workgroup included the CBPR tenet of incorporating stakeholder expertise and lived experience to inform research development. This approach can enable bidirectional engagement between community members and researchers related to research design and development (9, 20).

The importance of a CBPR approach is particularly salient for research with communities that historically have been marginalized, neglected, and/or subject to discrimination. Amongst indigenous communities, CBPR has been applied in a way that prioritizes shared ownership, cultural relevance, and tribal sovereignty (21), while addressing power imbalances between researchers and indigenous community members. The disability community represents another key example wherein CBPR approaches may address overcoming “ableism” in research and facilitating accessibility to research participation for disability community members. CBPR involving both of these two communities takes into account the unique historical, cultural, and present-day circumstances of these communities (22) to shift from research shaped by “Western” ideals to an indigenous or disability community directed research agenda (21). A similar approach was taken with the community stakeholder workshop in the present study, in which the research team borrowed aspects of grounded theory to enable stakeholders’ input regarding their respective communities to inform essential considerations for genetic screening research in diverse urban communities. This approach allows for the needs, priorities, and preferences of the community to emerge from community member stakeholders to shape the research, rather than solely relying upon researchers’ perceptions of community characteristics.

### 4.1 Limitations

The lack of inclusion of patients and community members without advocacy roles in this stakeholder workshop represents a primary limitation. This omission hinders the scope of experiences and perspectives that could inform genetic testing research development. For instance, no stakeholders mentioned the potential impact of language/linguistic factors, cultural beliefs, or use of findings for non-medical reasons, which could emerge as more salient discussion topics depending upon the composition of stakeholders. This highlights the need to expand the types of stakeholders included in future workshops.

The anonymization of stakeholder input presents another considerable limitation for several reasons. Although a range of stakeholder types was included (e.g., patient advocates, community partners, and PCPs), due to anonymization, we were unable to attribute comments and themes to stakeholder types. The aggregate reporting of stakeholder feedback prevented any examination of patterns in themes/responses across different stakeholder types; therefore, no conclusions could be drawn about whether certain concerns or priorities were predominately mentioned by a specific stakeholder type, nor whether they are representative of diverse communities. Similarly, the anonymized data prevented an examination of whether genetic condition ranking priorities differed by stakeholder type or specific communities. While a combination of methodologic barriers (e.g., reliance upon field notes) and an effort to establish initial rapport and trust amongst stakeholders supported the decision to anonymize stakeholder feedback, future research should carefully weigh this decision with the potential benefits of being able to attribute discussion input to stakeholders within a particular group. The inclusion of stakeholder types when presenting discussion themes would clarify the representativeness of the findings by providing the opportunity to attribute certain themes to specific types of stakeholders and diverse communities.

This stakeholder workshop was designed to address the specific priorities and perspectives of diverse communities within the Chicago metropolitan area. Although conclusions about representativeness cannot be made and *findings from stakeholder discussion* may be limited in generalizability to urban settings in the midwestern U.S., the *process* of convening a community stakeholder workshop as a step preparatory to the development of a community-based genetic testing program of research serves as an approach that may be modified and applied within a wide variety of community settings. Partnerships with community stakeholders are intended to be specific to the unique needs, priorities, and preferences of the community/ies in which the research will take place, which may pose inherent limitations to generalizability outside of these communities.

The approach to the prioritization of genetic conditions for testing was selected based on one of the aims of the workshop, which was to identify candidate genetic conditions to include in future research development. To achieve the goal of selecting four additional genetic conditions to include in testing alongside of the three CDC Tier 1 conditions, participants were asked to rank their top 4 conditions based on importance to include in the genetic testing research program with their responses analyzed using descriptive statistics. Future research would benefit from a more rigorous methodologic approach to ranking conditions, should the rankings be used to address research questions with a higher level of complexity.

### 4.2 Conclusion

To enhance the involvement of diverse communities in genetic research, effective community engagement throughout the research process is vital. The findings from this research development initiative demonstrated the benefits of a community engagement approach during the research design and development phase. Community stakeholders provided valuable input on the genetic conditions they considered most important, as well as considerations for research planning. There was clear consensus among stakeholders that trust is paramount to successful partnership with diverse communities to advance genetic research. Methodologic limitations preclude the ability to draw any conclusions about representation of community priorities because of the unknown stakeholder types associated with the perspectives reported. Ongoing community stakeholder engagement using a higher level of methodologic rigor is planned as a critical component to future phases of this initiative.

## Data Availability

The datasets presented in this article are not readily available because data were derived from field notes taken in real-time during a virtual workshop session that was not recorded or transcribed. Requests to access the datasets should be directed to sean.david@endeavorhealth.org.

## Footnotes

1
www.builduptrust.org

2
https://www.builduptrust.org/finalists-winners/sri-raj-lab

3
https://www.variantbio.com/research-partnerships
